# Association between growth differentiation factor‐15 (GDF‐15) with neurofilament light chain (NfL) and lower cognitive function with mediation from pulse pressure

**DOI:** 10.1002/alz70856_098746

**Published:** 2025-12-24

**Authors:** Kiat Sern Goh, Serena Low

**Affiliations:** ^1^ Changi General Hospital, Singapore, Singapore, Singapore; ^2^ Khoo Teck Puat Hospital (Alexandra Health Pte Ltd), Singapore, Singapore, Singapore

## Abstract

**Background:**

Growth differentiation factor‐15 (GDF‐15) is an emerging biomarker of mitochondrial dysfunction which increases oxidative stress and inflammation. Pulse pressure (PP) is a potential putative factor in cognitive impairment due to pulsatile strain that disrupts cerebral blood flow. We investigate the association between GDF‐15 with neurofilament light chain (NfL), a biomarker of neuroaxonal injury and cognitive function, with possible mediation by PP.

**Method:**

This was a cross‐sectional study of 100 participants recruited from health screening and specialist outpatient clinics in a tertiary hospital. Plasma GDF‐15 and NfL were measured with enzyme‐linked immunosorbent assay. Montreal Cognitive Assessment (MoCA) was used to assess cognitive function. The mediator was peripheral PP which was calculated from difference between systolic blood pressure and diastolic blood pressure.

**Result:**

The mean age was 55.6±12.9 years. The median GDF‐15 level was 692.4 (IQR 465.3‐911.6) pg/mL. GDF‐15 was positively correlated with NfL (correlation coefficient 0.520; *p* <0.001) and negatively correlated with MoCA score (correlation coefficient ‐0.213; *p* = 0.036). In linear regression, log‐transformed GDF‐15 was associated with higher NfL and lower MoCA score with adjusted coefficients 1.33 (95%CI 0.69,1.98; *p* <0.001) and ‐1.20 (95%CI ‐2.13, ‐0.26; *p* = 0.013) respectively. Mediation analysis showed that PP accounted for 15.7% of association between GDF‐15 and NfL (*p* = 0.046).

**Conclusion:**

GDF‐15 was associated with higher NfL and lower cognitive function in older individuals, with mediation from PP. GDF‐15 may play a potential role in cognitive impairment. These results may pave the way for future longitudinal studies and interventions targeting GDF‐15 and even PP in an effort to screen and prevent cognitive impairment.

